# Botulinum Toxin for Pain Relief in Cancer Patients: A Systematic Review of Randomized Controlled Trials

**DOI:** 10.3390/toxins16030153

**Published:** 2024-03-15

**Authors:** Lorenzo Lippi, Alessandro de Sire, Alessio Turco, Martina Ferrillo, Serdar Kesikburun, Alessio Baricich, Stefano Carda, Marco Invernizzi

**Affiliations:** 1 Physical and Rehabilitative Medicine, Department of Health Sciences, University of Eastern Piedmont “A. Avogadro”, 28100 Novara, Italy; alessio.turco.phys@gmail.com (A.T.); alessio.baricich@med.uniupo.it (A.B.); marco.invernizzi@med.uniupo.it (M.I.); 2 Department of Scientific Research, Campus LUdeS, Off-Campus Semmelweis University of Budapest, 1085 Budapest, Hungary; 3 Physical Medicine and Rehabilitation Unit, Department of Medical and Surgical Sciences, University of Catanzaro Magna Graecia, 88100 Catanzaro, Italy; alessandro.desire@unicz.it; 4 Research Center on Musculoskeletal Health, MusculoSkeletalHealth@UMG, University of Catanzaro “Magna Graecia”, 88100 Catanzaro, Italy; 5 Department of Health Sciences, University of Catanzaro “Magna Graecia”, 88100 Catanzaro, Italy; martina.ferrillo@unicz.it; 6 Ankara Gaziler Physical Therapy and Rehabilitation Education and Research Hospital, Department of Physical Medicine and Rehabilitation, Gulhane Medical School, University of Health Sciences Turkey, 06800 Ankara, Turkey; serdarkb@gmail.com; 7 Physical and Rehabilitation Medicine, “Ospedale Maggiore della Carità” University Hospital, 28100 Novara, Italy; 8 Neuropsychology and Neurorehabilitation Service, Department of Clinical Neuroscience, Lausanne University Hospital, 1004 Lausanne, Switzerland; stefano.carda@gmail.com; 9 Translational Medicine, Dipartimento Attività Integrate Ricerca e Innovazione (DAIRI), Azienda Ospedaliera SS. Antonio e Biagio e Cesare Arrigo, 15121 Alessandria, Italy

**Keywords:** botulinum toxin (BoNT), cancer, pain management, quality of life, rehabilitation

## Abstract

Cancer pain is one of the most disabling symptoms complained by cancer patients, with a crucial impact on physical and psychological well-being. Botulinum neurotoxins (BoNTs) type A and B have emerged as potential interventions for chronic pain; however, their role in these patients is still debated. Thus, this systematic review of randomized controlled trials aimed at assessing the effects of BoNT treatment for cancer pain to guide physicians in an evidence-based approach integrating BoNT in cancer care. Out of 5824 records, 10 RCTs satisfied our eligibility criteria and were included in the present work for a total of 413 subjects with several cancer types (breast, head and neck, esophageal, and thoracic/gastric cancers). While some studies demonstrated significant pain reduction and improved quality of life post-BoNT-A injections, outcomes across different cancer types were inconclusive. Additionally, several effects were observed in functioning, dysphagia, salivary outcomes, esophageal strictures, gastric emptying, and expansions. This review emphasizes the need for further standardized research to conclusively establish the efficacy of BoNT in comprehensive cancer pain management.

## 1. Introduction

Cancer pain is one of the most common symptoms experienced by cancer patients, representing a critical challenge in the rehabilitation management of these patients [1,2,3,4,5]. Due to its multifaceted nature, cancer pain is a complex experience involving sensory and emotional components, significantly impacting the physical, emotional, and social well-being of individuals with cancer [6,7,8,9,10,11].

In this context, pain might be associated with several causes including tumor compression or infiltration, surgery, chemotherapy, or radiation therapy, contributing substantially to the overall burden on quality of life and psychological well-being [12]. The WHO analgesic ladder offers a structured approach to managing cancer-related pain, designed to match the intensity of pain with appropriate medications [13,14]. On the other hand, recent research is now focusing on patient-reported outcomes, currently considered key elements in clinical decision-making for optimizing treatment outcomes [15]. In this scenario, its chronic nature and resistance to typical analgesic medications further complicate its management, underlining the need for effective treatments to reduce pain intensity and symptom burden [14].

Interestingly, botulinum neurotoxins (BoNTs) type A and B recently found extensive application in pain management in different pathological conditions [16]. Besides its clinical application being widely accepted in use in cosmetic treatments and neurological conditions [16,17,18,19], BoNT has also shown promise in managing neuropathic pain [16]. In particular, recent studies suggested BoNT might interact with nociceptive peripheral pathways, inflammation, and potentially with retrograde axonal transport towards the spinal cord [20,21]. In addition, a recent systematic review [16] underlined that integrating BoNT administration into the comprehensive management of pain could be effective not only in terms of pain intensity but also in improving multidimensional disability characterizing patients with chronic pain. 

Despite these positive findings, the implementation of BoNT in cancer pain is still debated. Recently, it has been proposed that local BoNT administration in cancer patients might reduce neuropathic pain and muscle spasms when injected near radiation or surgical sites, with long-term effects on pain intensity [22,23,24]. In accordance, both in vitro and in vivo studies have shown that BoNT might have a role in cancer growth, inducing cellular apoptosis and reducing tumor size [22,23,24,25]. On the other hand, a recent study [22] underlined a large gap of knowledge in this field, and systematic reviews with meta-analysis to conclusively establish the efficacy of botulinum toxin in cancer-related pain were lacking.

Therefore, this systematic review of randomized controlled trials (RCTs) aimed at assessing the effects of BoNT injections on pain relief in the comprehensive management of cancer patients, to guide clinicians in an evidence-based prescription of this intervention in the complex treatment framework of cancer patients.

## 2. Results

### 2.1. Study Characteristics

**Figure 1:**
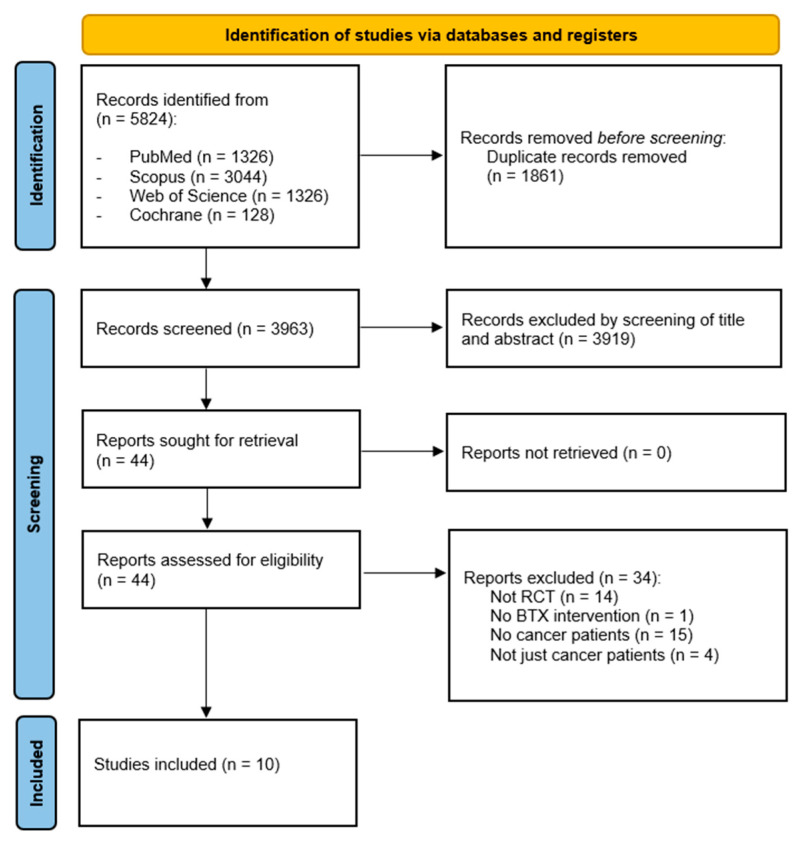
PRISMA 2020 flow diagram.

A total of 5824 records were identified across four databases. Following the removal of duplicates, 3963 studies underwent evaluation for eligibility based on title and abstract screening. Consequently, 3919 records were excluded, and 44 full-text studies underwent further evaluation. Of these, 34 articles were excluded due to inconsistency with eligibility criteria, and Supplementary Table S2 provides a detailed list of excluded studies along with the reasons for their exclusion. Lastly, 10 studies [26,27,28,29,30,31,32,33,34,35] were included in the qualitative synthesis. Further insights into the search process can be found in the PRISMA flow diagram shown in Figure 1. 

The studies included in this systematic review were published between 2006 [34] and 2023 [31]. Three studies were conducted in the USA [29,30,31], two studies were conducted in Belgium [27,28], two studies were conducted in Germany [32,34], one study was conducted in Iran [26], while the remaining two studies were carried out in China [33,35]. The sample size of the RCTs included ranged from 12 [32] to 78 [35], for a total of 413 subjects included in the systematic review.

The mean age of the subjects included ranged from 44.5 (range 27–64) years [29] to 66.3 ± 10.2 years [31].

The sample of the present study was composed of 153 patients with breast cancer [27,28,29,30], 55 patients with head and neck cancer [31,32,34], 145 patients with esophageal cancer [33,35], and 60 with thoracic and gastric esophageal cancer [26]. 

Body composition was characterized in three studies [27,28,35], with a mean BMI ranging from 23.35 ± 2.72 [35] to 28.1 ± 5.0 [27,28].

In all the studies (100%), the intervention was characterized by BoNT-A injections [26,27,28,29,30,31,32,33,34,35] and compared to different types of treatment. In particular, the control groups were treated with placebo infiltrations [27,28,29,30,31], a combination of BoNT-A and BoNT-B [32], high-dose of BoNT [34], triamcinolone acetonide [35], and only surgery procedures [26,33,35]. The characteristics of these studies are presented in detail in Table 1. 

### 2.2. Intervention Characteristics

In this systematic review, the intervention was characterized by injection of botulinum toxin A. Botulinum injections differ in dosage, posology, and injection site in relation to the type of cancer to be treated. In two studies [27,28] botulinum injections were combined with a physical therapy program.

#### 2.2.1. Intervention Characteristics Based on Cancer Type

All the intervention group was treated with BoNT-A injection. Based on the cancer type and the cancer treatment and/or procedures, the posology of the infiltrations was different.

− Breast cancer was assessed in four studies [27,28,29,30]. More in detail, in the studies conducted by De Groef et al. [27,28], the intervention was characterized by single BoNT-A infiltration combined with a standard physical therapy program. Intramuscular injection of BONT-A (100 units, OnabotulinumtoxinA; BOTOX, Allergan, Inc., Irvine, CA, USA) was administered in the pectoralis major muscle. Moreover, in the study by Gabriel et al. [29], a single injection of 40 units (20 units/mL) of onabotulinumtoxinA, was administered during surgery, in the pectoralis major muscle. In addition, a single injection of 100 units of botulinum toxin combined with 10 mL of normal saline throughout the pectoralis major muscle was performed and assessed in the study by Lo et al. [30].
− Head and Neck cancer was assessed in four studies [31,32,34]. More in detail, in the study conducted by Nieri et al. [31], the intervention was characterized by injection of 1 mL of onabotulinumtoxinA (50 units) in each submandibular gland. The injections were prepared by mixing 100 units of onabotulinumtoxinA in 2 mL of sterile saline in a syringe. Moreover, in the study by Teymoortash et al. [32], 15 U onabotulinumtoxinA was injected into the submandibular glands. In this study, there were two different intervention groups. In IG1, the BoNT-A was injected in the right gland, and NaCl (0.9%) in the left gland, while in the IG2, the BoNT-A was injected in the left gland, and NaCl (0.9%) in the right gland.

Wittekindt et al. [34] assessed the effects of low-dose BoNTA (abobotulinumtoxinA, Dysport, Ipsen Pharma, France) reconstituted in saline to a concentration of 10 mouse units (MU)/0.1 mL saline.

− Esophageal cancer was assessed in two studies [33,35]. More in detail, in the study conducted by Wen et al. [33], the intervention was characterized by a single session of BoNT-A (lanbotulinumtoxinA, Lanzhou Institute of Biological Products, Lanzhou, China) injections that was undertaken immediately after endoscopic submucosal dissection. A total of 100 units of BoNT-A was combined with 5 mL of saline solution (20 units/mL). The BoNT-A solution was injected in 0.5 mL increments into 10 separate points equally spaced along the circumference of the defect. Moreover, in the study by Zhou [35], lanbotulinumtoxinA was injected in 5 mL increments into 10 separate points at the level of the muscularis propria equally spaced along the circumference of the defect, immediately after the endoscopic submucosal dissection procedure. A total of 100 units of BoNT-A was combined with 5 mL of saline solution (20 units/mL).
− Thoracic and gastric esophageal cancer was assessed in one study [3]. More in detail, in the study conducted by Bagheri et al. [26], the intervention was characterized by a single injection of botulinum toxin into the pyloric sphincter muscle, immediately after surgery. OnabotulinumtoxinA was injected (200 units of toxin combined with 5 mL of 0.9% saline solution) with a 21 G needle at the upper and lower sections of the pyloric muscle in a transmural manner.

#### 2.2.2. Control Characteristics

− Placebo infiltrations were assessed in five studies [27,28,29,30,31]. In the two studies conducted by De Groef [27,28], the CG was treated with placebo (saline) infiltration. In particular, the placebo infiltration consisted of 50 mL saline (Mini-Plasco 20 mL B. Braun NaCl 0.9%). In the study by Gabriel et al. [29], the CG was treated with a single injection of 2 mL of NaCl during surgery, in the pectoralis major muscle. Moreover, in the study conducted by Nieri et al. [31], the CG was treated with a single injection of 1 mL of saline in each submandibular gland.
− A combination of BoNT-A and BoNT-B was assessed in one study [32]. In this study, control groups were treated with 15 U of onabotulinumtoxinA and 750 U BoNT-B (rimabotulinumtoxinB, Eisai Manufacturing Knowledge Centre, United Kingdom), injected into the submandibular glands. In particular, one control group was treated with BoNT-A and B in the right gland, and NaCl (0.9%) in the left gland, while the second control group was treated with BoNT-A and B in the left gland, and NaCl (0.9%) in the right gland.
− A high dose of BoNT was assessed by Wittekindt et al. [34]. This study assessed the effects of a low dose of onabotulinumtoxinA that was reconstituted in saline to a concentration of 10 mouse units (MU)/0.1 mL saline (intervention group). The control group was treated with a high dose of BoNT. In particular, BoNT-A was reconstituted in saline to a concentration of 20 MU/0.1 mL saline (high-dose group).
− Triamcinolone acetonide (TA) was assessed in the study conducted by Zhou et al. [35]. In particular, TA was combined with 0.9% NaCl to a final concentration of 4 mg/mL. A total of 40 mg (10 mL) TA was injected into the deep submucosa of the ulcer base at 10 sites, with a 1 mL dose at each site, immediately after the ESD procedure.
− Only surgery procedures were assessed in three studies [26,33,35]. No additional treatments were prescribed to the control groups.

### 2.3. Main Findings

#### 2.3.1. Pain Intensity

Pain intensity was assessed in four studies [27,29,30,34]. In particular, De Groef et al. [27] reported significant improvements in terms of the visual analogue scale (VAS 0–100) (*p* = 0.040) after single BoNT-A infiltration in breast cancer patients. According to the authors, there was a mean difference in change of 16 points on the VAS scale (0–100) for upper limb pain intensity, with a significant difference between groups favoring the intervention group (*p* = 0.040; 95% CI: 1 to 31). Nonetheless, when compared to the control group, the observed difference did not reach statistical significance (average change difference: 13/100; 95% CI: −4 to 31). Additionally, neither of the significant outcomes holds clinical relevance, as there is no meaningful decrease of at least 20/100 on the VAS. Furthermore, there were no discernible differences between groups in terms of pain quality at any given time point. There were no significant differences in pain prevalence rates at the upper limb (*p* = 0.754) and pectoral region (*p* = 0.258). In addition, in the study by Gabriel et al. [29], significant improvements, in terms of the VAS score, were reported in breast cancer patients treated with a single injection of BoNT-A, during surgery, in the pectoralis major muscle. There was a significant decrease in the pain level in the intervention group versus the placebo group (*p* < 0.0001). In the study conducted by Lo et al. [30], at any point throughout the postoperative period, there were no statistically significant differences in the mean change in pain levels (VAS score) between the breast cancer patients receiving botulinum toxin and the placebo group (all *p* > 0.05). Moreover, Wittekindt et al. [34] reported significant improvements in terms of pain in the VAS scale (*p* < 0.05) after BoNT-A injection (IG: low-dose; CG: high-dose). The low-dose group’s patients reported a statistically significant decrease in pain (VAS), from 4.3 on day 0 to 3.0 on day 28 (*p* < 0.05). The mean pain VAS values in the high-dose group did not improve significantly. Pressure hypersensitivity was assessed in one study [4]. More in detail, in the study by De Groef et al. [4], only for the serratus anterior muscle, the authors reported a significantly different change (0.61 kg/cm^2^; 95% CI: 0.07 to 1.15) after 1 month (*p* = 0.028).

Further details are shown in Table 1.

#### 2.3.2. Quality of Life

Quality of life was assessed in four studies [27,31,33,34]. De Groef et al. [27] reported significant improvements (*p* < 0.05) in terms of the quality of life assessed using Short Form Health Survey 36 (SF-36) after a single BoNT-A infiltration in breast cancer patients. Quality of life functional scales showed a borderline significant result for mental functioning domain was found in favor of the control group (*p* = 0.049). Moreover, the study by Nieri et al. [31] reported significant improvements in quality of life (*p* > 0.05) from V1 (1 week before radiation therapy) to V2 (1 week after radiation therapy) in both the control and onabotulinumtoxinA groups (IG: *p* = 0.049; CG: *p* = 0.034). In this study [31], the quality of life was assessed with the Oral Health Impact Profile-14 questionnaire (OHIP-14) to assess the quality-of-life outcomes and functional limitations caused by oral conditions. There was no significant improvement in OHIP-14 in either group from V2 to V3 (6 weeks after radiation therapy) or from V1 to V3 (*p* > 0.05). In addition, Wen et al. [33] reported significant improvements (*p* < 0.05) in patients with superficial esophageal carcinoma, in terms of the quality of life assessed by the Quality of Life Questionnaire (EORTCQLQ-OES18) after a single session of BoNT-A injections undertaken immediately after endoscopic submucosal dissection. The mean Quality of Life Questionnaire (EORTC QLQ-OES18) score was lower in the intervention group (25.8 ± 6.2) than in the control group (30.5 ± 7.2), and this difference was statistically significant (*p* < 0.05). Moreover, Wittekindt et al. [34] assessed quality of life by the Global Quality of Life (EORTC-QLQ-C-30) questionnaire after BoNT-A injection (IG: low-dose; CG: high-dose). A trend, but not a significant increase, in the global quality of life (*p* = 0.15) was reported in the low-dose group.

Further details are shown in Table 1.

#### 2.3.3. Physical Functioning

Functioning was assessed in two studies [27,28] to evaluate upper limb function, shoulder mobility, upper limb strength, shoulder statics and kinematics. In the study by De Groef et al. [27], shoulder function was assessed by disability of arm, shoulder, and hand (DASH) questionnaire; more in detail, no differences were found between the groups. There was a significant difference between the two groups for the prevalence rate of impaired shoulder function at one month (74% versus 96%, *p* = 0.096), favoring the intervention group. In the other study by De Groef et al. [28], shoulder function was assessed. No significant difference in the groups’ changes over time was observed for either the active forward or abduction range of motion (ROM). However, both groups showed an improvement in shoulder mobility. In the intervention and control groups, the prevalence rate of impairments for forward flexion decreased by 40% to 20% and 56% to 36%, respectively. A handgrip strength test was also used to measure upper limb strength. There was no significant difference between the groups in terms of changes in handgrip strength or in terms of prevalence rates of diminished handgrip strength over time. Moreover, the Acromion–Table Index, Pectoralis Minor Index, and scapular upward rotation were considered for shoulder statics and kinematics. Overall, no outcome measure showed statistically significant changes over time. There was just a significant difference in group changes from baseline to three months for scapular upward rotation at the upper limb’s maximum range of motion (>135°) (mean difference in change of 9° with 95% CI [0–9]).

Further details are shown in Table 1.

#### 2.3.4. Dysphagia and Salivary Outcomes

Salivary outcomes were assessed in two studies [31,32], and dysphagia was assessed in one study [33]. In particular, Nieri et al. [31] reported significant improvements in terms of salivary flow rate (mL/min) in the control group (saline injection) compared to the group treated with an injection of 1 mL of BoNT in each submandibular gland. In particular, in both groups, V2 (one week following radiation therapy) had a significantly reduced salivary flow rate than V1 (one week before radiation therapy) (*p* < 0.05). Six weeks after radiation therapy, there was no statistically significant difference in the salivary flow rates between V2 and V3 in either group (*p* > 0.05). On the other hand, the group that received BoNT treatment did not report a significant decrease in the overall salivary flow rate from V1 to V3 (*p* > 0.05), but the control group reported a significant reduction (*p* < 0.05). Moreover, in the study by Teymoortash et al. [32], salivary function was assessed by scintigraphy and salivary excretion fraction (SEF), and compared the effects of single BoNT-A injection versus NaCl injection, and BoNT-A combined with BoNT-B injection versus NaCl injection. There was no statistically significant difference in the scintigraphic uptake difference between BoNT and placebo, according to the analysis of the scintigraphic data (BoNT-A: *p* = 0.84 and BoNT-A-B: *p* = 0.56 for BoNT-A vs. placebo and BoNT-A-B vs. placebo, respectively). The authors also found no significant difference in treatment between BoNT and placebo in terms of salivary excretion fraction (BoNT-A: *p* = 0.44; BoNT-A-B: *p* = 0.44). The study conducted by Wen et al. [33] assessed dysphagia using the Mellow–Pinkas score. The Atkinson evaluation of dysphagia showed a significant difference between the two groups, favoring the BoNT group (*p* = 0.02).

Further details are shown in Table 1.

#### 2.3.5. Esophageal Strictures and Bougie Dilatations

Esophageal strictures were assessed in two studies [33,35], and bougie dilatations were assessed in one study [33]. In particular, Wen et al. [33] reported significant improvements in terms of stricture rate, and the number of dilatation procedures required by each patient for treatment of a stricture, after ESD followed by BoNT-A injection. the authors reported that there were significantly fewer patients in the intervention group (PP analysis, 6.1%, 2/33; ITT analysis, 11.4%, 4/35) than there were in the control group (PP analysis, 32.4%, 11/34; ITT analysis, 37.8%, 14/37) (*p* < 0.05) who suffered from esophageal stricture. Furthermore, there was a significant difference in the number of bougie dilation procedures between the intervention group (mean, 1.5; range, 0–2) and the control group (mean, 2.8; range, 0–5) (*p* < 0.05). Moreover, in the study conducted by Zhou et al. [35], the percentage of patients who developed stricture was 30.00% (intention to treat analysis, 9/30) and 26.92% (per protocol analysis, 7/26) in the BoNT-A group, 40.90% (intention to treat analysis, 9/22) and 43.75% (per protocol analysis, 7/16) in the triamcinolone acetonide group, and 84.21% (intention to treat analysis, 32/38) and 83.33% (per protocol analysis, 30/36) in the control group (*p* < 0.001). After examining the data of the two intervention groups, it was seen that the incidence of esophageal stricture was lower in the BoNT-A group (*p* < 0.001) and in the triamcinolone acetonide group (*p* = 0.004), compared to the control. In addition, the esophageal stricture in the entire circumference mucosal defect subgroup was considerably lower in the BoNT-A group compared to the triamcinolone acetonide group (33.3% vs. 100%, *p* = 0.0454).

Further details are shown in Table 1.

#### 2.3.6. Gastric Emptying

Gastric emptying was assessed in one study [26]. Bagheri et al. [26] did not report significant improvements in terms of gastric emptying, after a single injection of botulinum toxin into the pyloric sphincter muscle, immediately after esophagectomy. Delayed gastric emptying was determined by radiologists who performed the barium swallow tests under fluoroscopy. More in detail, the authors reported no significant difference between the two groups in terms of gastric emptying at 7 days (*p* = 0.446) and no significant differences after 3 weeks (*p* = 0.355).

Further details are shown in Table 1.

#### 2.3.7. Main Findings in Terms of Expansions

Amount of expansion and number of times to full expansion was assessed in one study [29]. Gabriel et al. [29] following a single BoNT injection, patients with breast cancer showed significant improvements in terms of volume expansions. More in detail, the authors reported that the volume of expansion each visit increased significantly in the intervention group compared to the placebo group (*p* < 0.0001).

Further details are shown in Table 1.

**Table 1:** Main characteristics of the studies included.

Authors and Year, *Journal*	Population							Intervention	Comparator	Protocol Duration	Outcomes	Main Findings
	Sample Size	Age (years)	BMI (kg/m^2^)	Male/Female	Cancer	Cancer Characteristics	Cancer Treatment					
Bagheri et al., 2013, *Asian Cardiovascular & Thoracic Annals*, Iran [26]	N = 60 IG = 30 CG = 30	N = 61 ± 10.7 (range 41–89)	NR	N = 33 M (55%)/27 F (45%) IG: 19 M (63.3%)/11 F (36.7%) CG: 14 M (46.6%)/16 F (53.4%)	Thoracic and gastric esophageal cancer	Esophageal cancer in the middle and lower third parts	Esophagectomy	After the surgery, a single injection of botulinum toxin was administered into the pyloric sphincter muscle. The injection consisted of 200 units of toxin combined with 5 mL of 0.9% saline solution, which was delivered using a 21 G needle. The injection was given transmurally, targeting both the upper and lower sections of the pyloric muscle.	No BoNT treatment	3 weeks follow-up)	− Gastric emptying − Discharge	This study reports about 60 patients with thoracic and gastric esophageal cancer (mean age: 61 ± 10.7; 55% male). Immediately after surgery, they were treated with single injection of botulinum toxin into the pyloric sphincter muscle. The study duration was 3 weeks (follow-up). The main finding is represented by no significant difference between the 2 groups in terms of gastric emptying at 7 days (*p* = 0.446) and no significant differences after 3 weeks (*p* = 0.355). The success rate of BoNT injection was 90%, and there were no negative effects.
De Groef et al., 2018, *Archives of physical medicine and rehabilitation*, Belgium [27]	N = 50 IG = 25 CG = 25	IG: 53.4 ± 10.0 CG: 56.6 ± 10.0	IG: 24.8 ± 3.6 CG: 28.1 ± 5.0	N = 50 F (100%)	Breast cancer	Primary breast cancer	− Mastectomy − Breast conserving − Mastectomy with immediate reconstruction − Axillary surgery − Radiotherapy − Chemotherapy − Neo-adjuvant chemotherapy − Immunotherapy − Endocrine treatment	Single Botulinum Toxin A (BoNT-A) infiltration + standard physical therapy program Intramuscular injection of BoNT-A (100 units, Allergan Botox) in the pectoralis major muscle. Standard physical therapy program of 12 weeks (one 30 min session per week).	Placebo (saline) infiltration + standard physical therapy program 50 mL saline (Mini-Plasco 20 mL B. Braun NaCl 0.9%). Standard physical therapy program of 12 weeks (one 30 min session per week).	6 months follow-up	− Pain − Quality of life − Shoulder function	This study reports about 50 breast cancer patients (mean age IG: 53.4 ± 10.0, CG: 56.6 ± 10.0; mean BMI IG: IG: 24.8 ± 3.6, CG: 28.1 ± 5.0; 100% female) They were treated with single injection of Botulinum Toxin A and standard physical therapy program. BoNT-A was injected into the pectoralis major muscle. The study duration was 6 months (follow-up). The main finding is represented by upper limb pain intensity. With a mean difference in change of 16 points on the VAS scale (0–100), there was a significant difference in upper limb pain intensity between the groups from baseline to 6 months, favoring the intervention group (*p* = 0.040; 95% CI: 1 to 31). For pain quality, no differences between groups were found. For pressure hypersensitivity only for the serratus anterior muscle a significantly different change was found (0.61 kg/cm^2^; 95% CI: 0.07 to 1.15) after 1 month (*p* = 0.028). There were no differences in upper limb function across the groups. There was a tendency to a significant difference between the two groups, with the intervention group showing a higher prevalence rate of reduced shoulder function at one month (74% versus 96%, *p* = 0.096). A marginally significant outcome (*p* = 0.049) in favor of the control group was obtained for the mental functioning domain in terms of quality of life.
De Groef et al., 2020, *European Journal of Cancer Care*, Belgium [28]	N = 50 IG = 25 CG = 25	IG: 53.4 ± 10.0 CG: 56.6 ± 10.0	IG: 24.8 ± 3.6 CG: 28.1 ± 5.0	N = 50 F (100%)	Breast cancer	Primary breast cancer	− Mastectomy − Breast conserving − Mastectomy with immediate reconstruction − Axillary surgery − Radiotherapy − Chemotherapy − Neo-adjuvant chemotherapy − Immunotherapy − Endocrine treatment	Single Botulinum Toxin A (BoNT-A) infiltration + standard physical therapy program Intramuscular injection of BoNT-A (100 units, Allergan Botox) in the pectoralis major muscle. Standard physical therapy program of 12 weeks (one 30 min session per week).	Placebo (saline) infiltration + standard physical therapy program 50 mL saline (Mini-Plasco 20 mL B. Braun NaCl 0.9%). Standard physical therapy program of 12 weeks (one 30 min session per week).	6 months follow-up	− Active shoulder range of motion − Upper limb strength − Scapular statics and kinematics	This study reports about 50 breast cancer patients (mean age IG: 53.4 ± 10.0, CG: 56.6 ± 10.0; mean BMI IG: IG: 24.8 ± 3.6, CG: 28.1 ± 5.0; 100% female) They were treated with single injection of Botulinum Toxin A and standard physical therapy program. BoNT-A was injected into the pectoralis major muscle. The study duration was 6 months (follow-up). There has only been a significant difference (*p* = 0.042) in the change between groups from baseline to three months for scapular upward rotation during maximal range motion (>135°) of the upper limb (mean difference in change of 9°; 95% CI: 0 to 9). However, both groups had overall improvements in shoulder function and range of motion with the physical therapy for active forward flexion.
Gabriel et al., 2015, *Aesthetic Surgery Journal*, USA [29]	N = 30 IG = 15 CG = 15	N = 44.5 (range 27–64)	NR	N = 30 F (100%)	Breast cancer	NR	Unilateral or bilateral tissue expander Breast reconstruction following therapeutic skin-sparing or nipple-sparing mastectomy	Single injection of 40 units (20 units/mL) of neurotoxin (OnabotulinumtoxinA; BOTOX, Allergan, Inc., Irvine, CA, USA), during surgery, in the pectoralis major muscle	Single injection of 40 units (20 units/mL) of placebo (0.9% NaCl), during surgery, in the pectoralis major muscle	12–36 months follow-up	− Pain − Amount of expansion − Number of times to full expansion − Safety	This study reports about 30 breast cancer patients (mean age: 44.5 [range 27–64]; 100% female) They were treated with single injection of Botulinum Toxin A, during surgery. BoNT-A was injected into the pectoralis major muscle. The study duration was 12–36 months (follow-up). The VAS score showed a significant difference (*p* < 0.05) between the two groups, indicating reduced pain in the IG. Furthermore, a noteworthy rise in the volume of expansion each visit was seen in the intervention group vs. to the placebo group (*p* < 0.0001). The usage of the neurotoxic did not result in any adverse effects.
Lo et al., 2015, *Annals of Plastic Surgery*, USA [30]	N = 23	N = 46.5 (range 28–68)	NR	N = 23 F (100%)	Breast cancer	NR	Bilateral mastectomy	100 units of botulinum toxin were injected in a fan-like manner throughout the pectoralis major muscle after tion with 10 mL of normal saline.	100 units of normal saline placebo were injected throughout the pectoralis major muscle	12 weeks follow-up	− Pain − Complications	This study reports about 23 breast cancer patients (mean age 46.5 [range 28–68]; 100% female) They were treated with single injection of Botulinum Toxin A. BoNT was injected into the in the pectoralis major muscle. The study duration was 12 weeks (follow-up). At every stage of the postoperative period, there were no statistically significant improvements in the mean change in pain levels between the botulinum toxin and placebo groups (all *p* > 0.05). The complication rate did not differ significantly between the groups (*p* = 0.53).
Nieri et al., 2023, *Head & Neck*, USA [31]	N = 20 IG = 10 CG = 10	IG: 55.1 ± 10.2 CG: 66.3 ± 10.2	NR	N = 33 M (55%)/27 F (45%) IG: 7 M (70%)/3 F (30%) CG: 8 M (80%)/2 F (20%)	Head and neck cancer	Stage III or IV head and neck squamous cell carcinoma	Radiation or chemoradiation	Injection of 1 mL of BoNT (50 units) in each submandibular gland (100 units of BoNT were mixed in 2 mL of sterile saline in a syringe)	Injection of 1 mL of 1 mL of saline in each submandibular gland (2 mL of sterile saline in a syringe)	6 weeks follow-up	− Quality of Life − Salivary flow − Safety	This study reports about 20 head and neck cancer patients (mean age IG: 55.1 ± 10.2, CG: 66.3 ± 10.2; 55% male) They were treated with single injection of Botulinum Toxin A. BoNT-A was injected in each submandibular gland. The study duration was 6 months (follow-up). The main finding is that both the control and BoNT groups resulted in a significant improvement in quality of life from V1 (one week prior to radiation therapy) to V2 (one week following radiation therapy) (IG: *p* = 0.049; CG: *p* = 0.034). The OHIP-14 did not significantly improve in either group from V1 to V3 or from V2 to V3 (6 weeks after radiation therapy) (*p* > 0.05). Salivary flow rate was significantly reduced in both groups in V2 (one week following radiation therapy) compared to V1 (one week prior to radiation therapy) (*p* < 0.05). Six weeks following radiation therapy, there was no statistically significant difference in the salivary flow rates between V2 and V3 in either group (*p* > 0.05). The control group resulted in a significant decrease in salivary flow rate from V1 to V3 (*p* < 0.05), but the group that received BoNT treatment did not experience this same reduction (*p* > 0.05). At V3, the BoNT group’s level of the neutrophil chemoattractant CXCL-1 (GRO) was lower than that of the control group.
Teymoortash et al., 2016, *PLoS One*, Germany [32]	N = 12 IG1 = 3 IG2 = 3 IG3 = 3 IG4 = 3	N = 55.4 (range 45–66)	NR	N = 10 M (83%)/2 F (17%)	Head and neck cancer	Stage III or IV head and neck squamous cell carcinoma	Chemoradiation	15 U BoNT/A (Allergan Pharmaceuticals, Ireland) was injected into the submandibular glands. IG1: BoNT/A injected in the right gland, NaCl (0.9%) in the left gland. IG2: BoNT/A injected in the left gland, NaCl (0.9%) in the right gland	15 U BoNT/A and 750 U BoNT/B (Eisai Manufacturing Knowledge Centre, United Kingdom) were injected into the submandibular glands. IG3: BoNT/A and B in the right gland, NaCl (0.9%) in the left gland IG4: BoNT/A and B in the left gland, NaCl (0.9%) in the right gland	6 months follow-up	− Salivary function − Safety and tolerability − Total amount of radioactivity within the gland	This study reports about 12 head and neck cancer patients (mean age: 55.4 [range 45–66]; 83% male) The study duration was 6 months (follow-up). The main finding is represented by significant improvements in the scintigraphic uptake difference. The scintigraphic uptake difference between BoNT and placebo was not significantly different, according to the analysis of the scintigraphic data (BoNT/A: *p* = 0.84 and BoNT/A-B: *p* = 0.56 for BoNT/A vs. placebo and BoNT/A-B vs. placebo, respectively). Additionally, the salivary excretion fraction did not significantly differ between the BoNT and placebo groups (BoNT/A: *p* = 0.44; BoNT/A-B: *p* = 0.44). The BoNT/A and B were well tolerated.
Wen et al., 2016, *Gastrointestinal Endoscopy*, China [33]	N = 67 IG = 33 CG = 34	N = 60.4 ± 11.4 IG = 61.8 ± 7.9 CG = 60.1 ± 7.3	NR	N = 46 M (68.7%)/21 F (31.3%) IG: 23 M (69.7%)/10 F (30.3%) CG: 23 M (67.6%)/11 F (32.4%)	Esophageal carcinoma	Esophageal cancer in the upper, middle, and lower third parts	Endoscopic submucosal dissection	BoNT-A injections were administered after ESD. 100 units of BoNT-A were combined with 5 mL of saline solution and injected into 10 points around the circumference of the defect in 0.5 mL increments.	No BoNT treatment	12 weeks	− Quality of Life − Dysphagia − Esophageal stricture − Bougie dilation	This study reports about 67 esophageal cancer patients (mean age IG: 61.8 ± 7.9; CG = 60.1 ± 7.3; 68.7% male) They were treated with single injection of Botulinum Toxin A. BoNT-A was injected into 10 separate points equally spaced along the circumference of the defect immediately after endoscopic submucosal dissection. The study duration was 12 weeks (follow-up). The main finding is represented by significant improvements in the Quality of Life. The Group B had a mean EORTC QLQ-OES18 score of 30.5 ± 7.2, while group A had a mean score of 25.8 ± 6.2. This difference was statistically significant (*p* < 0.05). The Atkinson assessment of dysphagia showed a significant difference (*p* = 0.02) between the two groups, in favor of BoNT group. In the intervention group, there were significantly less patients diagnosed with esophageal stricture (PP analysis, 6.1%, 2/33; ITT analysis, 11.4%, 4/35) than in the control group (PP analysis, 32.4%, 11/34; ITT analysis, 37.8%, 14/37) (*p* < 0.05). Moreover, there was a statistically significant difference in the number of bougie dilation procedures between the intervention group (mean, 1.5; range, 0–2) and control group (mean, 2.8; range, 0–5) (*p* < 0.05).
Wittekindt et al., 2006, *Laryngoscope*, Germany [34]	N = 23 IG1 = 13 IG2 = 10	N = 60.4 ± 11.4	NR	N = 21 M (91.3%)/ 2 F (8.7%)	Head and neck cancer	Stage II–III squamous cell carcinoma of the upper aerodigestive tract	Radical cervical neck dissection	BoNT-A formulation Dysport (Ipsen Pharma, Ettlingen, Germany). BoNT-A was reconstituted in saline to a concentration of 10 mouse units (MU) per 0.1 mL saline (low-dose group).	BoNT-A formulation Dysport (Ipsen Pharma, Ettlingen, Germany). BoNT-A was reconstituted in saline to a concentration of 20 MU/0.1 mL saline (high-dose group).	28 days	− Pain − Quality of Life	This study reports about 23 head and neck cancer patients (mean age 60.4 ± 11.4; 91.3% male) They were treated with single injection of Botulinum Toxin A. BoNT-A was injected into the submandibular glands. The study duration was 28 days (follow-up). The main finding is represented by significant improvements in pain. Patients in the low-dose group showed a statistically significant decrease in pain (VAS) from 4.3 on day 0 to 3.0 on day 28 (*p* < 0.05). The high-dose group’s pain VAS scores did not significantly improve. In the low-dose group, there was a trend in global quality of life, but not a statistically significant increase (*p* = 0.15). There were no adverse events observed.
Zhou et al., 2021, *Journal of Cancer*, China [35]	N = 78 IG1 = 26 IG2 = 16 CG = 36	IG1: 65.15 ± 7.23 IG2: 65.06 ± 7.88 CG: 65.28 ± 8.11	IG1: 23.35 ± 2.72 IG2: 23.47 ± 3.04 CG: 23.83 ± 3.03	IG1: 65.4% M/34.6% F IG2: 62.5% M/37.5 F CG: 72.2% M/17.8% F	Esophageal cancer		Endoscopic submucosal dissection	IG1: BoNT-A solution was injected into 10 points at the muscularis propria level immediately after the ESD procedure. Each injection was done in 5-mL increments, and a total of 100 units of BoNT-A were used. The solution was combined with 5 mL of saline solution, resulting in a concentration of 20 units/mL.	IG2: TA mixed with NaCl was injected into the submucosa of the ulcer base at 10 sites (1 mL per site) after the ESD procedure, totaling 40 mg (10 mL). CG: No BoNT treatment	12 weeks	− Esophageal strictures	This study reports about 78 esophageal cancer patients (mean age IG1: 65.15 ± 7.23, IG2: 65.06 ± 7.88, CG: 65.28 ± 8.11; IG1: 65.4% male, IG2: 62.5% male, CG: 72.2% male) They were treated with single injection of Botulinum Toxin A. BoNT-A was injected into 10 separate points at the level of the muscularis propria equally spaced along the circumference of the defect, immediately after ESD procedure. The study duration was 12 weeks (follow-up). The main finding is represented by significant improvements in esophageal stricture development. In the BoNT-A group, the percentage of patients developing stricture was 30.00% (intention to treat analysis, 9/30) and 26.92% (per protocol analysis, 7/26). In the TA group, it was 40.90% (intention to treat analysis, 9/22) and 43.75% (per protocol analysis, 7/16). In the control group, the percentage was 84.21% (intention to treat analysis, 32/38) and 83.33% (per protocol analysis, 30/36) (*p* < 0.001). Subsequent analysis comparing the two groups revealed that the incidence of esophageal stricture was reduced in the BoNT-A group (*p* < 0.001) and in the TA group (*p* = 0.004). In addition, the esophageal stricture in the BoNT-A group was significantly lower compared to the TA group in the entire circumference mucosal defect subgroup (33.3% vs. 100%, *p* = 0.0454).

Abbreviations: BMI: body mass index; CG: control group; CXCL-1 (GRO): inflammatory cytokine CXCL-1 (GRO); EORTC QLQ-OES18: Quality of Life Questionnaire; IG: intervention group; NR: not reported; OHIP-14: Oral Health Impact Profile-14; TA: triamcinolone acetonide; VAS: visual analog scale.

### 2.4. Meta-Analysis 

According to the *Cochrane Handbook for Systematic Review of Intervention* [36], a meta-analysis was performed only for homogeneous studies in terms of samples, interventions, and outcomes.

**Figure 2:**
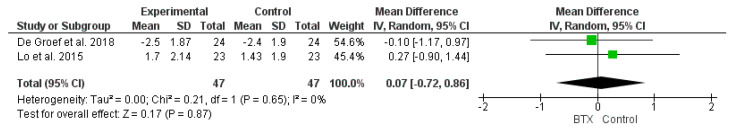
Forest plot comparing the BoNT-injection group to control in terms of mean change in pain intensity (VAS) at 1 month after intervention [27,30]. The mean change was calculated as difference between 1-month assessment and pre-operative assessment (baseline).

In our comprehensive meta-analysis, we examined the mean change in perceived pain at different post-intervention time points. Only two studies were included in the analysis [27,30]. The initial focus was on the 1-month follow-up, assessing the difference between baseline measurements and assessments conducted one month after the initiation of the study, specifically before the injection of BoNT. The analysis did not show significant improvements in mean difference (MD) for perceived pain [ES: 0.07 (−0.72, 0.86), *p* = 0.87]. Figure 2 shows further details of the meta-analysis. 

**Figure 3:**
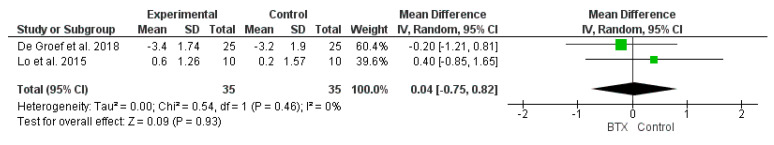
Forest plot comparing the BoNT-injection group to control in terms of mean change in pain intensity (VAS) at 3 months after intervention [27,30]. The mean change was calculated as difference between 3-month assessment and pre-operative assessment (baseline).

Moreover, a parallel analysis investigating the 3-month post-intervention follow-up period did not show significant improvements in MD for pain intensity, with an effect size (ES) of 0.04 (−0.75, 0.82) and a *p*-value of 0.93. See Figure 3 for further details. 

### 2.5. Quality Assessment and Risk of Bias

According to the Jadad scale [37], nine studies (90%) of the RCTs included [27,28,29,30,31,32,33,34,35] were of high quality, while only one was of low quality [26]. Table 2 shows the score of each subitem of the Jadad scale for the RCTs included in detail.

Points were assigned based on the following criteria: 1 point for a study described as randomized, 1 point for appropriate randomization, 1 point for subjects blinded to the intervention, 1 point for the evaluator blinded to the intervention, and 1 point for a description of withdrawals and dropouts.

**Figure 4:**
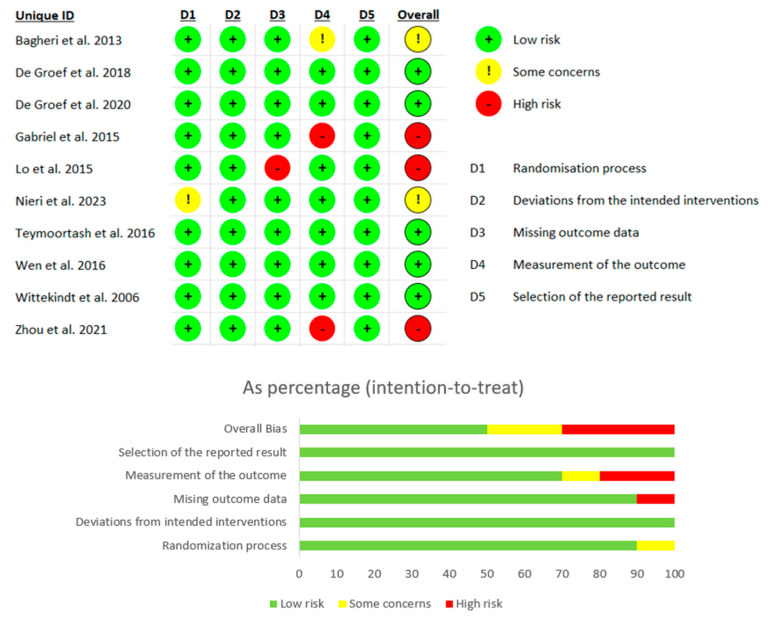
Risk of bias of the included studies [30,31,32,33,34,35,36,37,38,39] according to RoBv.2.

The risk of bias was assessed by RoBv.2 [38]. The process showed that nine studies (90%) [26,27,28,29,30,32,33,34,35] ensured correct randomization, while one study [31] showed some concerns in this item due to baseline group differences. Three studies (30%) [26,29,35] showed some concerns [26] or high risk of bias [29,35] in the fourth domain due to the lack of details about the blinding of the study operators and assessors. Lo et al. [30] showed a high risk of bias in the missing outcome data domain, due to the decrease in patients assessed at different time point of the study. All studies (n = 10, 100%) showed a low risk of bias in the deviations from the intended interventions domain [26,27,28,29,30,31,32,33,34,35] (see Figure 4)

## 3. Discussion

Despite growing evidence underlining the potential role of BoNT in the management of chronic pain, its effects in cancer pain management are far from being fully elucidated. Thus, this systematic review of RCTs with meta-analysis provided a broad overview about the current evidence supporting BoNT injection in cancer pain management. 

Our findings showed that BoNTs have been studied in different cancer types, including breast cancer, head and neck cancer, esophageal cancer, and thoracic/gastric esophageal cancer. While BoNT-A is the most studied, a large heterogeneity of administration modalities has been proposed. More in detail, BoNT-A injections, were administered in different dosages and sites based on the specific cancer type and treatment procedures. 

On the other hand, it is not surprising that conflicting results were reported in terms of pain intensity. In particular, De Groef et al. [28] reported significant improvements in VAS scores post-BoNT-A infiltration in breast cancer patients, suggesting potential implications for an integrated treatment implementing the functional recovery of breast cancer patients. In contrast, Lo et al. [30] did not identify significant differences between intervention and control group. These conflicting data might be related in the different administration modalities and different therapeutic interventions. 

On the other hand, our meta-analysis did not show significant improvements in terms of pain intensity. However, it should be noted that this analysis included only two studies due to the heterogeneity of study participants, treatment protocols, and outcome measures. The lack of significant improvements in perceived pain observed at 1 month [ES: 0.07 (−0.72, −0.86), *p* = 0.87] raises doubts about integrating BoNT-A injections in the pain management strategies in cancer pain. Despite these considerations, the lack of significance might reflect the slight number of study available and considering the strong evidence supporting the positive effect of BoNT injection in pain management [16,39], further studies are needed to clarify the role of BoNT in cancer pain. 

On the other hand, no significant effects were reported after 3 months [ES: 0.04 (−0.75, 0.82), *p* = 0.93]. These results might be partly related to the pharmacokinetics of BoNT molecules that might start to reduce its effects after 3 months from the injection [40]. Besides these considerations, long-term solutions are frequently not possible in cancer pain refractory to conventional therapies [41], and BoNT injections might be considered a potential treatment option in specific patients with neuropathic pain. 

Moreover, it should be noted that breast cancer survivors have several comorbidities (e.g., osteoporosis, oral diseases, lymphedema, etc.), and the injection procedures should be considered as a part of a wider comprehensive rehabilitation aimed to reduce pain and to improve HR-QoL [9,42,43]. In this context, pain is considered one of the most common symptom reported by cancer patients significantly affecting their quality of life [44]. Around 20–60% of patients with breast cancer and approximately 30% of those with head and neck cancer might be affected by chronic pain specifically at the area where radiation or surgery occurred [45]. Thus, it is crucial integrating the most effective strategies in cancer pain management aiming at reducing pain intensity and its effect in emotional and psychological sphere. On the other hand, the results of the present work are in line with recent systematic reviews supporting the multidimensional effects of BoNT-A in pain management [16,39]. 

In accordance, our findings suggested positive insights also in terms of quality of life of cancer patients with four studies different RCTs addressing this topic. Despite these considerations, a quantitative synthesis of data reported was not possible due to the differences of study participants and the different outcome scales used to assess quality of life (SF-36 [28], OHIP-14 scores [31], EORTC-QLQ-C-30 [34], and EORTC QLQ-OES18 [33]).

Therefore, to the best of our knowledge, the present work represents the first systematic review of RCTs addressing the role of BoNT-A in cancer pain, summarizing the current evidence on BoNT administration protocols in different cancer types. The results of the present work might improve knowledge about this topic although different works have assessed this topic without a systematic way. Interestingly, the preview review by Mittal and Jabbari [22] supported the effects of BoNT-A in neuropathic pain and local muscle spasm management in cancer patients. However, the authors assessed both animals and human studies without focusing on RCTs. As a result, strong evidence supporting BoNT-A implications in clinical management of cancer pain are lacking. 

Similarly, the recent scoping review by Suraj et al. [46] focused on the therapeutic effects of BoNT-A in malignant psoas syndrome, reporting promising results of integrating BoNT injections in the comprehensive management of this painful and disabling condition. Despite the strict eligibility criteria and the rigid methodology of literature review, the authors included case report and case series, making difficult to draw strong conclusions about the effects of the studies interventions. Lastly, the meta-analysis by Awadeen et al. [47] underlined the potential benefits of BoNT injections in breast surgeries. On the other hand, the authors did not focus on patients with breast cancer and the therapeutic effects mainly focused on aesthetic outcomes, while the effects of pain intensity remain to be fully characterized. 

Thus, this is the first systematic review of RCTs assessing the role of BoNT in cancer pain management. Our findings might promote a deep understanding of the therapeutic effects of specific BoNT injection procedures and could be considered as a catalyst for the development of specific therapeutic intervention targeting the complex framework underpinning refractory cancer pain.

Despite these considerations, this systematic review is not free from limitations. More in detail, the large heterogeneity in cancer types represents the main limitation of this study. However, we included different cancer types to provide a comprehensive overview of the different natures of pain, with potential implications for understanding the different mechanisms and manifestations of pain in distinct malignancies. In order to address this issue, we deeply characterized the different interventions based on specific cancer types, providing a broad overview of the current literature in a systematic way. This approach not only enhances the granularity of our analysis but also acknowledges the challenges in cancer pain management. In addition, it should be noted that only two studies were included in the meta-analysis to guarantee a good quality for the quantitative synthesis. 

These limitations reflect the large gap of knowledge in this field and call for further good-quality studies clarifying the role of BoNT injection in cancer pain management.

## 4. Conclusions

To the best of our knowledge, this is the first systematic review of RCTs assessing the role of BoNT in pain management of cancer pain. The RCTs analyzed in this systematic review have shown promising results in terms of pain relief and HR-QoL. These findings suggest that BoNT could effectively alleviate symptoms in individuals experiencing cancer pain with intriguing implication for a comprehensive multimodal approach of this disabling condition. On the other hand, several BoNT protocols have been proposed in the current literature, emphasizing the need for a better standardization to comprehensively understand the most effective and cost-efficient strategies for BoNT injections in pain management. Thus, future good quality studies are crucial to draw strong conclusions that can guide clinicians in implementing precise treatments to enhance the comprehensive pain management in cancer patients.

## 5. Materials and Methods

### 5.1. Registration

The Preferred Reporting Items for Systematic Reviews and Meta-analyses (PRISMA) statement [48] was followed in conducting this systematic review. A preliminary search was performed for similar review protocols in progress in the International Prospective Register of Systematic Reviews (PROSPERO). No similar review was found; thus, the systematic review was accepted on 7 December 2023 (PROSPERO protocol number CRD42023486695). 

### 5.2. Search Strategy

Two independent investigators simultaneously conducted a systematic search of four databases (PubMed/Medline, Scopus, Web of Science, and Cochrane Central Register of Controlled Trials (CENTRAL)) to identify published randomized controlled trials until 20 September 2023. The search strategies for each database are shown in Supplementary Table S1.

### 5.3. Selection Criteria

In accordance with the PICO model [49], we considered eligible randomized controlled trials (RCTs) satisfying the following eligibility criteria:

− (P) Participants: patients over 18 years old with cancer.
− (I) Intervention: pre-operative, intra-operative, or post-operative treatment with botulinum.
− (C) Comparator: any comparator.
− (O) Outcome: The primary outcome was pain intensity. Secondary outcomes were physical functioning, fatigue, and quality of life.

Only RCTs published in peer-reviewed international journals were considered. The exclusion criteria were as follows: (i) studies involving animals; (ii) participants experiencing pregnancy, clinical instability, or palliation; (iii) master’s or doctorate theses and conference proceedings; (iv) studies in languages other than English. There was no restriction on publication date during the database search.

The retrieved records were assessed for duplication via automated tools, and the resulting papers were screened by two independent investigators, reviewing the titles and abstracts, excluding the papers that did not meet the inclusion criteria. Any disagreement was discussed involving a third reviewer to reach consensus. Two independent reviewers evaluated the eligibility of the resulting studies in full text, and pertinent data were extracted using Microsoft Excel 365 (version 2402). Any discrepancies were resolved through discussion between the two reviewers or by seeking the input of a third reviewer.

### 5.4. Data Extraction and Synthesis

The extracted data included the following details: (1) title; (2) authors; (3) publication year; (4) nationality; (5) participant information (including number, mean age, age range, and gender); (6) characteristics of interventions; (7) comparator; and (8) main findings.

The data were extracted from full-text documents by two authors independently. Any disagreement between the two reviewers was solved by collegial discussion among the authors. In case of disagreement, a third author was asked. Text and tables were used to provide a descriptive summary and explanation of study characteristics and findings.

### 5.5. Quality Assessment and Risk of Bias 

Two authors independently evaluated the quality of the included RCTs using the Jadad scale [37]. Discrepancies were resolved through collegial discussion within the author team. The Jadad scale comprises five items, with a total score ranging from zero to five. The assessed items included (a) random sequence generation; (b) appropriate randomization; (c) blinding of participants or personnel; (d) blinding of outcome assessors; (e) withdrawals and dropouts. RCTs achieving a Jadad score between three and five points were classified as high quality.

The Cochrane risk-of-bias tool for randomized trials (RoBv.2) [38] was used to assess the risk of bias. Based on the RoBv.2 items, bias was graded as low, high, or uncertain. RoBv.2 specifically analyzed the following domains: (i) random process; (ii) deviation from the intended interventions; (iii) missing outcome data; (iv) measurement of the outcome; and (v) selection of the reported result.

## Figures and Tables

**Table 2:** Quality assessment of the studies included in the present systematic review.

Articles	Domain					Score
	Random Sequence Generation	Appropriate Randomization	Blinding of Participants or Personnel	Blinding of Outcome Assessors	Withdrawals and Dropouts	
Bagheri et al. 2013 [26]	1	0	0	0	1	2
De Groef et al. 2018 [27]	1	1	1	1	1	5
De Groef et al. 2020 [28]	1	1	1	1	1	5
Gabriel et al. 2015 [29]	1	1	0	0	1	3
Lo et al. 2015 [30]	1	1	1	1	1	5
Nieri et al. 2023 [31]	1	1	1	1	1	5
Teymoortash et al. 2016 [32]	1	1	1	1	1	5
Wen et al. 2016 [33]	1	1	1	1	1	5
Wittekindt et al. 2006 [34]	1	0	1	1	1	4
Zhou et al. 2021 [35]	1	1	0	0	1	3

## Data Availability

The data presented in this study are available on request from the corresponding author.

## Supplementary Materials

The following supporting information can be downloaded at: https://www.mdpi.com/article/10.3390/toxins16030153/s1, Supplementary Table S1: Search Strategy; Supplementary Table S2: Record excluded.
