# Supplementary material for: Botulinum Toxin for Pain Relief in Cancer Patients: A Systematic Review of Randomized Controlled Trials
Source: Toxins (Basel). 2024 Mar 15;16(3):153. doi: 10.3390/toxins16030153 (PMC10974124; doi:10.3390/toxins16030153)
Supplement: Supplementary file 1 [file toxins-16-00153-s001.zip › Table S1. Search strategy (1).pdf]

Table S1. Search strategy.

PubMed:

("botulinum toxins"[MeSH Terms] OR ("botulinum"[All Fields] AND "toxins"[All Fields]) OR "botulinum toxins"[All Fields] OR ("botulinum"[All Fields] AND "toxin"[All Fields]) OR "botulinum toxin"[All Fields] OR "BTX"[All Fields] OR ("botulinum toxins"[MeSH Terms] OR ("botulinum"[All Fields] AND "toxins"[All Fields]) OR "botulinum toxins"[All Fields] OR ("botulinum"[All Fields] AND "neurotoxins"[All Fields]) OR "botulinum neurotoxins"[All Fields]) OR ("botulinum toxins"[MeSH Terms] OR ("botulinum"[All Fields] AND "toxins"[All Fields]) OR "botulinum toxins"[All Fields] OR ("clostridium"[All Fields] AND "botulinum"[All Fields] AND "toxins"[All Fields]) OR "clostridium botulinum toxins"[All Fields]) OR ("botuline"[All Fields] OR "botulinic"[All Fields] OR "botulinum toxins"[MeSH Terms] OR ("botulinum"[All Fields] AND "toxins"[All Fields]) OR "botulinum toxins"[All Fields] OR "botulin"[All Fields])) AND ("cancer s"[All Fields] OR "cancerated"[All Fields] OR "canceration"[All Fields] OR "cancerization"[All Fields] OR "cancerized"[All Fields] OR "cancerous"[All Fields] OR "neoplasms"[MeSH Terms] OR "neoplasms"[All Fields] OR "cancer"[All Fields] OR "cancers"[All Fields] OR "carcinoma"[MeSH Terms] OR "carcinoma"[All Fields] OR "carcinomas"[All Fields] OR "carcinoma s"[All Fields] OR "cysts"[MeSH Terms] OR "cysts"[All Fields] OR "cyst"[All Fields] OR "neurofibroma"[MeSH Terms] OR "neurofibroma"[All Fields] OR "neurofibromas"[All Fields] OR "tumor s"[All Fields] OR "tumoral"[All Fields] OR "tumorous"[All Fields] OR "tumour"[All Fields] OR "neoplasms"[MeSH Terms] OR "neoplasms"[All Fields] OR "tumor"[All Fields] OR "tumour s"[All Fields] OR "tumoural"[All Fields] OR "tumourous"[All Fields] OR "tumours"[All Fields] OR "tumors"[All Fields])

Records: 1326

Scopus:

TITLE-ABS-KEY (((botulinum AND toxin) OR (Botulinum AND Neurotoxins) OR (Clostridium AND botulinum AND Toxins) OR (Botulin) OR (BTX)) AND ((cancer) OR (neoplasms) OR (carcinoma) OR (tumor)))

Records: 3044

Web of Science:

ALL= ((botulinum toxin OR BTX OR Botulinum Neurotoxins OR Clostridium botulinum Toxins OR Botulin) AND (Cancer OR Neoplasm OR Carcinoma OR Tumor))

Records: 1326

Cochrane:

ID	Search
#1	MeSH descriptor: [cancer] explode all trees
#2	MeSH descriptor: [neoplasm] explode all trees

#3	MeSH descriptor: [tumor] explode all trees
#4	MeSH descriptor: [carcinoma] explode all trees
#5	MeSH descriptor: [botulinum toxin] explode all trees
#6	MeSH descriptor: [BTX] explode all trees
#7	MeSH descriptor: [Botulinum Neurotoxins] explode all trees
#8	MeSH descriptor: [Clostridium botulinum Toxins] explode all trees
#9	MeSH descriptor: [Botulinum] explode all trees
#10	(#1 OR #2 OR #3 OR #4) AND (#5 OR #6 OR #7 OR #8 OR #9)

Records: 128
