# Supplementary material for: Botulinum Toxin for Pain Relief in Cancer Patients: A Systematic Review of Randomized Controlled Trials
Source: Toxins (Basel). 2024 Mar 15;16(3):153. doi: 10.3390/toxins16030153 (PMC10974124; doi:10.3390/toxins16030153)
Supplement: Supplementary file 1 [file toxins-16-00153-s001.zip › Table S2. Record excluded (1).pdf]

Table S2. Characteristics of excluded studies assessed in full-text.

Study	*Reason for exclusion*
Abedini et al. 2020	No cancer patients
Attal et al. 2016	No cancer patients
Bach et al. 2012	Not RCT
Disphanurat et al. 2021	No cancer patients
Dougles et al. 2002	Not RCT
Eckardt et al. 2002	Not RCT
Figus et al. 2009	Not RCT
Ginsberg et al. 2012	No cancer patients
Grise et al. 2010	No cancer patients
Hackert et al. 2016	No cancer patients
Hartl et al. 2008	Not RCT
Hartl et al. 2008 (2)	Not RCT
Keaney et al. 2019	No cancer patients
Kumagai et al. 2014	No cancer patients
Kuo 2005	Not RCT
Laskawi et al. 2013	Not RCT
Layeeque et al. 2004	Not RCT
Lee et al. 2017	No BTX intervention
Lee et al. 2021	No cancer patients
Lee et al. 2021 (2)	No cancer patients
Lemaine et al. 2020	Not just cancer patients
Ma et al. 2021	Not just cancer patients
Mittal et al. 2012	Not RCT
Naderinabi et al. 2017	No cancer patients
Naik et al. 2015	No cancer patients
Puentes-Gutiérrez et al. 2021	Not RCT
Richardson et al. 2000	Not just cancer patients
Rostami et al. 2016	Not RCT
Stewart et al. 2017	Not RCT
Verghese et al. 2020	No cancer patients

Wittekindt et al. 2004	Not RCT
Yaraghi et al. 2018	No cancer patients
Zelken et al. 2015	Not just cancer patients
Zirovich et al. 2021	No cancer patients
